# Etiological associations of cerebral venous sinus thrombosis among adult sudanese patients: a multicenter cross-sectional study

**DOI:** 10.1097/MS9.0000000000000650

**Published:** 2023-04-14

**Authors:** Mohamed Malekaldar, Khabab Abbasher Hussien Mohamed Ahmed, Yassin Abdalla, Mohammed Eltahier Abdalla Omer, Amira Siddig, Khalid Mohammed, Mazin S. Hassan Haroun, Abbasher Hussien, Mohammed Mahmmoud Fadelallah Eljack

**Affiliations:** a Omdurman Teaching Hospital; b Faculty of Medicine, University of Khartoum; c Faculty of Medicine, Omdurman Islamic University; d Faculty of Medicine, Al Neelain University, Khartoum; e Faculty of Medicine and Health Sciences, Gadarif University, Al Qadarif; f Department of Community, University of Bakht Alruda, Ad Duwaym, Sudan

**Keywords:** cerebral venous sinus thrombosis, cerebral venous thrombosis, CVST, stroke

## Abstract

Cerebral venous sinus thrombosis (CVST) is a common condition that, if not detected and treated promptly, carries a high risk of morbidity and mortality. CVST’s most common aetiological associations are post-partum, pregnancy, and oral contraceptive pills. The purpose of this study was to look into the aetiology of CVST in Sudanese patients in neurological centres in Khartoum state.

**Methodology:**

This was a cross-sectional hospital bases study conducted on CVST patients at four neurological centres in Khartoum state in the period from March to October 2020. Patients were studied for the aetiological association of CVST using a standardized questionnaire including medical history, clinical examination, investigation, and treatment.

**Results:**

The study included about 60 patients, 50 of whom were female (83.3%) and 10 of whom were male (16.7%). In terms of clinical presentation, almost all patients had headache, followed by visual disturbances in 49 (81.7%), seizures in 46 (76%), disturbed consciousness in 12 (20%), and weakness in 12 (20%). The most common sign was abnormal speech, which was found in eight patients (13.3%), memory disturbances in eight patients (13.3%), evidence of CN VI lesion in three (5%), papilledema in 49 (81.7%), and hemiparesis in 46 (76.7%), while abnormal sensory signs were found in only one patient. The most common aetiological association were pregnancy in 15 (25%), oral contraceptive pills in 11(18.3%), and being in the post-partum period in 23(38.3%). All of the patients’ magnetic resonant imaging/magnetic resonant venography results were abnormal. Six patients had extensive sinus involvement, 35 had superior sagittal sinus involvement, and 19 had transverse sinus involvement. After treatment, 45 patients (75%) fully recovered, 11 (18.3%) partially recovered, and 4 (6.7%) died.

**Conclusion:**

Post-partum, pregnancy, and oral contraceptive pills were the most common aetiological associations of CVST compared with other populations.

## Introduction

Highlights

- Cerebral venous sinus thrombosis (CVST) is a common condition that, if not detected and treated promptly, carries a high risk of morbidity and mortality.
- In this study, post-partum, pregnancy, and oral contraceptive pills were the most common aetiological associations of CVST.
- Headache and papilledema had the highest occurrence in our patients.
- Superior sagittal sinus thrombosis and transverse sinus thrombosis were the most common types of CVST.
- We recommend advising and encouraging the women regarding the early mobility after the delivery.

CVST is an infrequent cause of stroke, representing 0.5% of all cerebrovascular events on a global scale^1^. Despite the disease being uncommon from a global perspective, CVST is of particular interest in low-income countries which bear the heaviest burden of the disease. CVST is particularly common in young and middle-aged groups, with females demonstrating the highest frequency of occurrence. This extremely skewed sex distribution is likely a product of the sex-specific risk factors of oral contraceptives, pregnancy, and puerperium^2^.

Predisposing causes of cavernous venous thrombosis (CVT) are multiple, with at least one risk factor implicated in 85% of affected adults. Furthermore, multifactorial causes have been identified in a substantial number of cases while in small proportion of cases remain idiopathic^3,4^. Prothrombotic states, either genetically imposed or acquired, represent a large component of the aetiology. Deficiencies of Antithrombin III, protein C, and protein S along with factor V Leiden mutation (resistance to activated protein C) among genetic thrombophilias, are well-established predisposing conditions. Women who take oral contraceptives and have the prothrombin G20210 mutation may be at particularly high risk for CVST^5,6^. Acquired prothrombotic states such as antiphospholipid antibodies have also been implicated in the development of CVST. Malignancy, systemic inflammatory conditions, haematological conditions, dehydration, and infections among other precipitants, can exacerbate hypercoagulability and provoke CVT in susceptible patients. Additionally, manoeuvres that may directly or indirectly alter cerebral venous circulation have also been identified as risk factors for CVST in hospital settings^7,8^.

The extensive and highly variable spectrum of clinical presentation makes it particularly difficult for diagnosis of CSVT, and a high index of clinical suspicion is required for detecting affected individuals. Neuroimaging studies are therefore of paramount importance, as venous sinus occlusion is readily visualized using MR or computed tomography venography or, conventional X-ray angiography. Guidelines from both the European Stroke Organization and the American Heart Association outlined anticoagulation with a therapeutic dose of heparin as the primary treatment for CVT, regardless of the presence of intracranial haemorrhage at baseline^9,10^. Longer-term Oral anticoagulation therapy with vitamin K antagonists follows the acute treatment, although the optimal duration of antithrombotic therapy has not been established by randomized controlled studies. Anticoagulation is often continued indefinitely if thrombophilia is diagnosed. Direct oral anticoagulants have not been validated in the treatment of CVT. Similarly, surgical approaches to CVT have no proven benefit; mechanical thrombectomy is therefore utilized as a lifesaving measure in selected refractory cases.

The natural course of CVST differs significantly from the various subtypes of arterial stroke, with the overall outcome being affected by the patient’s age, anatomical location of the involved sinus and associated cerebral veins, and the involvement of the cerebral parenchyma. Around 4% of mortality exists in the acute phase, usually due to complications of cerebral oedema, coma, and herniation^11^. Patients have an increased risk of developing sinovenous thrombosis recurrence or other forms of venous thromboembolism in about one-third of cases^12^.

In the field of aetiology, it should be considered that a large portion of CVT takes place in African and Asian countries, where obstetric causes are responsible for up to 30% of the incidence. The relevance of cultural factors will probably increase soon as a consequence of the rapid demographic changes induced by migration flows. The second consequence of the important role of OC and pregnancy-puerperium as causative factors is that it fully qualified CVT as a gender-related neurological disorder.

The signs and symptoms of CVT are highly variable and often change with the stage of the disease. Analysis of the scientific literature demonstrates a paucity of studies that specifically assess the clinical presentation and temporal evolution of symptoms. Headache has been identified as the most frequent presenting symptom, but so far, no specific features have been identified. Since headache is one of the most frequent conditions observed in the emergency setting, the identification of patients with CVT and headache as an isolated symptom is a challenging task. New case-control studies specifically designed to assess the problems of the clinical presentation and of the differential diagnosis of CVT, with a special attention to headache are, therefore, urgently needed^11^.

New imaging techniques have considerably eased the diagnosis of CVT; the current gold standard is magnetic resonant imaging (MRI) of the brain with venous MRI angiography. However, these techniques still have a low sensitivity in the case of isolated thrombosis of a cortical vein or early sinus thrombosis; in these instances, conventional angiography is needed. The choice of the most appropriate and accurate examination must be based on the clinical features.

The best available therapy for CVT is currently early anticoagulation with heparin. An interesting alternative in the acute setting is offered by local thrombolysis, but its use is currently not supported by scientific evidence and should be considered experimental. Any delay in the diagnosis and treatment of CVT can have dramatic clinical consequences: venous thrombosis is a time-dependent process whose progress may be stopped by anticoagulants.

Overall, the best clinical management of CVT depends on the optimization of three main steps: the identification of clinical indicators that give rise to a diagnostic hypothesis in the early stage of the disease, the choice of the best radiologic examinations to support this suspicion and the early institution of the appropriate treatment^13^.

In this study, we aimed to describe the aetiological associations of CVST among Sudanese patients, identify the risk factors of CVST in Sudanese patients, to correlate between clinical features, and the findings of the brain imaging of CVT patients and assess the outcome of management of CVST.

## Materials and methods

### Study setting

This was a cross-sectional hospital-based study included all patients diagnosed with and admitted to Neurology centers in Khartoum State, Sudan, over the period of 8 months from March 2020 to October 2020. Those younger than 18 years and those who refused to participate were excluded.

This Study has been reported in line with the STROCSS criteria^14^.

### Sample size

Our sampling method was the total coverage of all patients fulfilling the study criteria. The number was 60 patients.

### Data collection

Data were collected using a structured standardized interviewer-administered questionnaire, consisting of detailed history (including personal data, symptoms, and signs of CVT, past medical history, family history, and social history), in addition to investigation results and treatment.

Clinical examination was done for all patients and included: general examination, and a central nervous system examination looking for evidence of cerebral venous thrombosis.

The following investigations were done for each patient: complete blood count, random blood glucose, liver function test, renal function test, international normalization ratio, thrombophilia screening, and connective tissue disease screening.

Brain magnetic resonant imaging and magnetic resonant venography (MRV), brain computed tomography and brain computed scan venography were done for some patients.

### Data analysis

Data was entered into the computer from a master sheet using a software program. It was entered and analyzed in the Statistical Package for Social Sciences (SPSS 26).

### Ethical considerations

The proposal was presented to the ethics review committee of the Sudan Medical Specialization Board, Council of MD Neurology for approval of the study.

All patients were adult Sudanese patients (defined as the age 18 years or more). All patients gave their consent to participate in the study. The participants were aware of their rights throughout the study; participation in the study was completely voluntary and confidentially considered.

Written approved consent was taken from the concerned hospitals.

## Results

Sixty patients with cerebral venous thrombosis were included in this study, 50 of them (83.3%) were females and 10 (16%) were males. Nine patients (15%) were aged 18–25 years, 45 (75%) aged 26–50, and 6 (10%) aged more than 50. Thirty seven patients (61.7%) resided in Khartoum State and 23 (38.3%) in other states. Two patients (0.03%) were single, 49 (81.7%) were married, 3 (5%) were widowed, and 6 (10%) were divorced. Regarding occupation, 3 patients (5%) were office employees, 5 (8.3%) were labourers, 51 (85%) were housewives, and 1 (1.7%) was retired (Table 1).

**Table 1:** Demographic characteristics

Demographic characteristics	Frequencies	Percentage
Sex		
Male	10	16.7
Female	50	83.3
Marital status		
Single	2	3.3
Married	49	81.7
Widow	3	5
Divorced	6	10
Age group		
18–25	9	15
26–50	45	75
>50	6	10
Occupation		
Employee	3	5
Labourer	5	8.3%
W/H	51	85
Retired	1	1.7%

W/H, house wife.

Regarding clinical presentation, headache was found in all patients, seizures in 46 (76%), disturbed consciousness in 12 (20%), abnormal behaviour in 9 (15%), visual disturbances in 49 (81.7%), diplopia in 3 (5%), fever in 7 (11.7%), neck stiffness in 2 (3.3%), weakness in 12 (20%) (Table 2).

**Table 2:** The distribution of the study participants according to the clinical symptoms (*n*=60)

	Frequency	Percent
Headache	60	100.0
Seizures	46	76.7
Visual disturbances	49	81.7
Abnormal behaviour	9	15.0
Weakness	12	20.0
Fever	7	11.7

Regarding the clinical examination signs abnormal speech was found in eight patients (13.3%), memory disturbances in 8 (13.3%), evidence of CN VI lesion in 3 (5%), papilledema in 49 (81.7%), hemiparesis in 46 (76.7%), paraparesis in 6 (10%), quadriparesis in 2 (11.7%), and monoparesis in 1 (1.7%). Abnormal sensory signs were only found in one patient (Table 3).

**Table 3:** The distribution of the study participants according to clinical signs (*n*=60)

	Frequency	Percent
Disturb LOC	12	20
Memory disturbance	8	13.3
Speech disturbance	8	13.3
Cranial nerve VI involvement	3	5.1
Papilledema	48	80
Neck stiffness	2	3.3
Motor weakness	12	20
Sensory disturbance	1	1.7

LOC, loss of consciousness.

**Figure 1:**
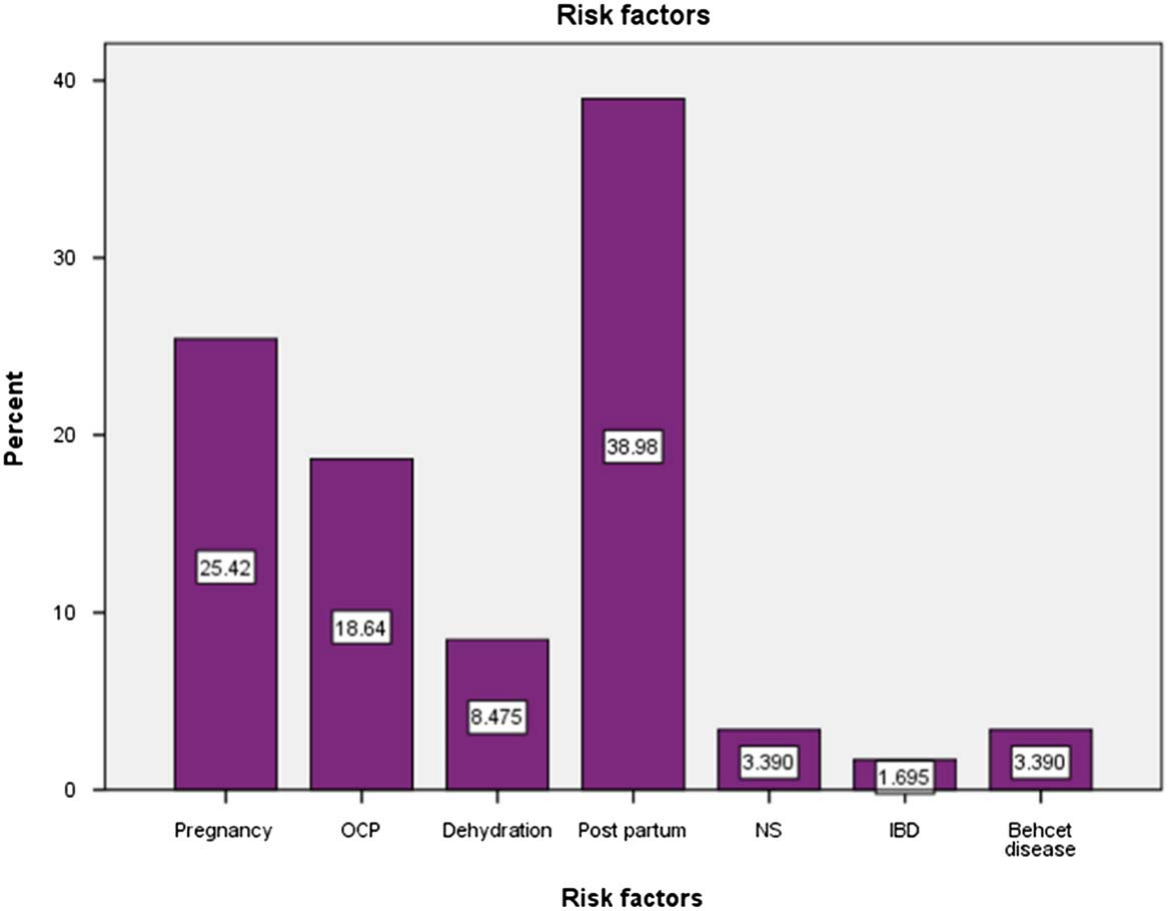
The distribution of the study participants according to the risk factors (*n*=60). IBD, inflammatory bowel diseases; NS, nephrotic syndrome; OCP, oral contraceptive pills.

Post-partum period was a risk factor in 23 patients (38.3%), pregnancy in 15 (25%), oral contraceptive pills (OCP) in 11 (18.3), dehydration in 5 (8.3%), nephrotic syndrome in 2 (3.3%), and IBD in 1 (1.7%). Regarding past medical history: Hypertension was detected in 1 (1.7%) patient, malignancy in 3 (5%), rheumatologic and connective tissue disease in 1 (1.7%), and skin disease in 1 (1.7%) (Table 4 and Figure 1).

**Table 4:** The distribution of the study participants according to the risk factors (*n*=60)

	Frequency	Percent
Pregnancy	15	25.0
OCP	11	18.3
Dehydration	5	8.3
Post-partum	23	38.3
NS	2	3.3
Malignancy	3	5
CTD	1	1.7
Total	60	100

CTD, connective tissue disease; NS, nephrotic syndrome; OCP, oral contraceptive pills

The distribution of the study participants according to the type of intracranial pressure reduction therapy received was made (Table 5).

**Table 5:** The distribution of the study participants according to the type of intracranial pressure reduction therapy received (*n*=60)

	Frequency	Percent
Acetazolamide	49	81.6
Intervention therapy (LP shunt)	2	3.4
Repeated lumbar puncture	9	15

LP, lumbar puncture.

Abnormalities found in the investigation results were as follows: complete blood count in three (5%) patients, erythrocyte sedimentation rate in 2 (3.3%), RBS in 2 (3.3%), prothrombin time, renal function test, and liver function test were normal in all patients. antinuclear antibodies was abnormal in 1 (1.7%) patient, anti-DNA in 1 (1.7%), lupus antibody in 1 (1.7%), anticardiolipin antibody in 1 (1.7%), protein C in 3 (5%), protein S in 3 (5%). Antithrombin III, B20 210MAB gene screening, and Factor V Leiden screening were requested for seven patients but were not done for the others. MRI/MRV was abnormal in all the patients. Extensive sinus involvement was found in six patients, the superior sagittal sinus in 35 patients, and the transverse sinus in 19 patients (Table 6).

**Table 6:** The distribution of the study participants according to the type of venous sinus thrombosis (*n*=60)

	Frequency	Percent
Extensive sinus involvement	6	10
Superior sagittal sinus	35	58.3
Transverse sinus	19	31.6

Low-molecular-weight heparin was administered to 2 patients (3.3%), warfarin to 26 (43.3%), and rivaroxaban to 26 (43.3%). Intracranial pressure management was done on all patients. Acetazolamide was administered to 49 patients, intervention therapy (LP shunt) to 2, and repeated lumbar puncture to 9. Forty-five patients (75%) fully recovered after treatment, but 11 (18.3%) partially recovered and 4 (6.7%) died (Table 7).

**Table 7:** The distribution of the study participants according to the outcome (*n*=60)

	Frequency	Percent
Full recovery	45	75.0
Partial recovery	11	18.3
Death	4	6.7
Total	60	100.0

## Discussion

We found that post-partum, pregnancy, and OCP were the most common aetiological associations of CVST compared with other populations. Females were more common than males in the study. Headache and papilledema had the highest occurrence in our patients. Superior sagittal sinus thrombosis and transverse sinus thrombosis were the most common types of CVST in our study. Most of our patients in this study had good outcomes^3^. CVT often occurs in young people and is much less in comparison with arterial stroke. CVST should be considered in the differential diagnosis of any patients who have risk factors for CVST and share symptoms and signs of other neurological diseases at presentation. Because CVST is a treatable condition and has good outcomes, the early detection of patients and performing suitable workup is essential. Clinical characterization according to interpretations of patients having CVST can contribute to developing a set of attributes that may be used for the investigation and clinical evaluation of patients. Our study included 60 patients with CVST assessed for the aetiological associations and showed that most of the patients were young females and the mean age was found to be 35 years. As mentioned in the literature, poor outcome was associated with age older than 37 years, male sex, mental status disorder, coma, intracranial haemorrhage on admission, cancer, central nervous system infection, and thrombosis of the deep cerebral venous system^3^. CSVT Clinical findings can be due to two main mechanisms; Occlusion in cerebral veins or occlusion of venous sinuses^11^. The clinical features showed that all of our patients suffered from headaches, and the majority of them had seizures, visual disturbances, and focal neurological deficits, and 46% revealed papilledema. This is compatible with the results mentioned in the literature^7,15,16^. Concerning headache in CVST, it is the presenting clinical symptom in more than 85% of CVST patients^3^. The headache could be due to the stretching of dura which is innervated by branches of the trigeminal nerve, or diffuse stretching of dura due to raised ICP^17^. Concerning Seizures, it’s a known consequence of CVST. Acute seizures are often associated with focal neurological deficits and are correlated with Post‐CVST epilepsy^18^. Papilledema is common in the presentation of CVST and was found in a study to be the initial presentation in 54% of patients with a time of resolution of almost 6 months^19^. Our study revealed that post-partum, pregnancy, and usage of OCP were found to be more associated risk factors. This is compatible with what was mentioned in the literature. Hormonal changes, especially during pregnancy and post-partum, make women 3 times more prone to acquire CVST^3^.

In a multicenter study of 465 women having CVST, 17% happened during puerperium and pregnancy^20^. Pregnancy and post-partum women with CVST may have a good prognosis in the long term, as in in a study, all 11 patients during Partum or post-partum had a better outcome at 6 months follow-up (*P* < 0.05)^21,22^. CVST in pregnancy and post-partum women with CVST can cause extreme outcomes such as death, as in a study where death was the outcome in 11% (*n*=8/73)^22,23^. Another known risk factor for CVST among women is OCP use and misuse, and the latter can be considered an even worse risk factor as the risk of getting CVST can be two times higher^24^. Summary Odds ratio for developing CVST was 5.59 in women using OCP in comparison with controls (95% CI 3.95–7.91; *P*<0.001) in the pooled analysis of 17 studies^25^. Some of the patients with CVST had nephrotic syndrome, CA breast, and connective tissue disease (SLE). In patients with a history of antiphospholipid antibodies or nephrotic syndrome; a careful evaluation for Acquired thrombophilia should be done. More risk factors for CVST include chronic inflammatory diseases; inflammatory bowel disease, systemic lupus erythematosus, vasculitis, and malignancy^13^. Our study revealed that some patients had a low level of protein C and S; this is similar to what was mentioned in the pieces of literatures. Other than pregnancy, post-partum, and usage of OCP, we can find CVST in other conditions that lead to a prothrombotic state including genetic and acquired thrombophilia. Genetic thrombophilia includes factor V Leiden mutation, prothrombin gene mutation *20210*, protein C and protein S deficiencies, and hyperhomocysteinemia, in addition to antithrombin deficiency^13^. It did appear that all patients with CVST had abnormal imaging including Brain MRI, and MRV. Due to the higher sensitivity, MRI is considered the gold standard for diagnosis of CVST over computed tomography. Our study showed that superior sagittal sinus thrombosis and transverse sinus thrombosis were more common types of CVST; this is similar to an International study where the frequency of the sites of CVST was as follows:

1. Transverse sinus 86%
2. Superior sagittal sinus 62%.
3. Straight sinus 18%.
4. Cortical veins 17%.
5. Jugular veins 12%.
6. Vein of Galen and internal brain veins 11%^3^.

Concerning management of CVST, the initial approach focuses on treating life-threatening complications of CVST, especially seizures, increased intracranial pressure, and coma. Intracranial pressure reduction therapy was administered to all of our patients, with the majority receiving acetazolamide, and only a few receiving repeated LP, and LP shunt. All of our patients received anticoagulation, including low-molecular-weight heparin, and oral anticoagulation (rivaroxaban or warfarin). Our study showed that most treated patients of CVST had a good outcome. Anticoagulants are the standard treatment for cerebral venous thrombosis. Low-molecular-weight heparin is recommended in the guidelines^9^. Anticoagulants are used in patients already having a thrombus or prone to forming additional thrombi. They are used to prevent thrombus propagation preventing further complications of CVST such as pulmonary embolism and deep venous thrombosis. Thrombolysis is indicated in patients who do not improve with anticoagulation therapy^13^.

One of the limitations for this study is the relatively small sample size, hence further studies with a large sample size is recommended, we also recommend advising and encouraging the women with the workers in these sectors (Obstetricians and Gynaecologists, midwives, and general practitioners) regarding the early mobility after the delivery; emphasizing the importance of knowing the side effects of oral contraceptive use particularly those at high risk with avoidance of tight circumcision as much as possible to increase post-partum mobility. Healthcare systems should be alarmed to follow strategies to control improper use of OCP. We should consider the possibility of CVST in any abnormal finding in the image that is not compatible with arterial territories. We must do a full workup for thrombophilia screening and connective tissue diseases, because the disease tends to recur, and the patients may need lifelong treatment. Because the visual insult is common in CVST with a nasty sequel, ophthalmological and neurosurgical consultation has paramount importance. Further studies in this field are mandatory.

## Conclusion

This study revealed that post-partum; pregnancy and OCP were the most common aetiological associations of CVST compared with other populations. Females were more common than males in the study. Headache and papilledema had the highest occurrence in our patients. Superior sagittal sinus thrombosis and transverse sinus thrombosis were the most common types of CVST in our study. Most of our patients in this study had good outcomes.

## Ethical approval

The proposal was presented to the ethics review committee of the Sudan Medical Specialization Board, Council of MD Neurology for approval of the study.

## Consent

All patients were adult Sudanese patients (defined as the age 18 years or more). All patients gave their consent to participate in the study. The participants were aware of their rights throughout the study; participation in the study was completely voluntary and confidentially considered. written approved consent was taken from the concerned hospitals.

## Source of funding

This research did not receive any specific grant from funding agencies in the public, commercial, or not-for-profit sectors.

## Author contribution

All authors participated in planning the study, data collection, results, and discussion sections.

## Conflicts of interest disclosure

The authors declare that they have no competing interests.

## Research registration unique identifying number (UIN)

1. Name of the registry: None.
2. Unique Identifying number or registration ID: None.
3. Hyperlink to your specific registration (must be publicly accessible and will be checked): None.

## Guarantor

Mohammed Mahmmoud Fadelallah Eljack.

## Provenance and peer review

Not commissioned, externally peer-reviewed.

## Availability of data and materials

The materials datasets used and/or analyzed during this study are available from the corresponding author on reasonable request.
